# Data of the life cycle impact assessment and cost analysis of prospective direct recycling of end-of-life reverse osmosis membrane at full scale

**DOI:** 10.1016/j.dib.2020.106487

**Published:** 2020-11-05

**Authors:** Jorge Senán-Salinas, Junkal Landaburu-Aguirre, Alberto Blanco, Raquel García-Pacheco, Eloy García-Calvo

**Affiliations:** a IMDEA Water Institute, Avenida Punto Com. n °2. 28805, Alcalá de Henares, Madrid, Spain; b Chemical Engineering Department, University of Alcalá, Ctra. Madrid-Barcelona Km 33.600, 28871, Alcalá de Henares, Madrid, Spain; c Laboratory of Chemical and Environmental Engineering (LEQUIA), Institute of the Environment, University of Girona, Girona, 17003, Spain

**Keywords:** Life Cycle Assessment, Geographic Information Systems, Circular Economy, Reverse Osmosis

## Abstract

This data includes the geographical data, the Life Cycle Inventory data and Life Cycle Assessment data of the implementation of end-of-life (EoL) reverse osmosis (RO) direct recycling implementation at full scale in a Spanish region. Besides, the data allows the comparison of the environmental profile between recycled membrane products with the commercial counterparts. The EoL-RO stock potential was analysed constrained to the Segura´s watershed. However, the distribution of recycled membranes was considered within the European Union´s borders. The International Life Cycle Data system (ILCD) midpoint impact categories and the indicator Service Life Ratio (SLR) are presented. This data could be used for deepening analyses as the externalities monetarisation or business model identification or policymakers

This data article is related to J. Senán-Salinas, A. Blanco, R. García-Pacheco, J. Landaburu-Aguirre, E- García-Calvo. J Prospective Life Cycle Assessment and economic analysis of direct recycling of end-of-life reverse osmosis membranes based on Geographic Information Systems. J. Clean. Prod. In Press

**Specifications Table**

**Table:** 

Subject	Environmental Engineering
Specific subject area	Life Cycle Assessment and Geographic Information Systems
Type of data	Tables / Graphs/ Figures/ Datasets
How data were acquired	Inventories were obtained from real experimentation, GIS databases, literature and databases (Ecoinvent v3.4). Life Cycle Impact Assessment was estimated with the ILCD method. Transport Costs were obtained from ACOTRAM v3.1 database.
Data format	Raw /Analysed /Filtered
Description of data collection	Geographical information. Mass and energy flows from real experimentation, literature and LCA databases (Ecoinvent v3.4.). Other specific databases as AEDYR database for desalination facilities.
Data source location	Spain
Data accessibility	Repository name: Mendeley Data DOI: http://dx.doi.org/10.17632/z6db5w8d6k.1 URL: https://data.mendeley.com/datasets/z6db5w8d6k/1
Related research article	J. Senán-Salinas, A. Blanco, R. García-Pacheco, J. Landaburu-Aguirre, E- García-Calvo. J Prospective Life Cycle Assessment and economic analysis of direct recycling of end-of-life reverse osmosis membranes based on Geographic Information Systems. J. Clean. Prod. *In Press* DOI: https://doi.org/10.1016/j.jclepro.2020.124400

**Value of the Data**

• The impact results with different functional units allow the comparison of the overall impact of of the full implementation of recycling strategies. The spatial information will allow further analysis of logistics within membrane direct recycling. Montecarlo Life cycle Inventory results allows the reproducibility of the study.
• Those results could be used for researchers focus on logisctics and membrane recycling. As well as researchers focused on economic, circular economy transition and policy making.
• The data could be used for externalities quantification and monetarisation, reproduce the research and develop further logistic analysis.

## 1 Data Description

**Fig. 1:**
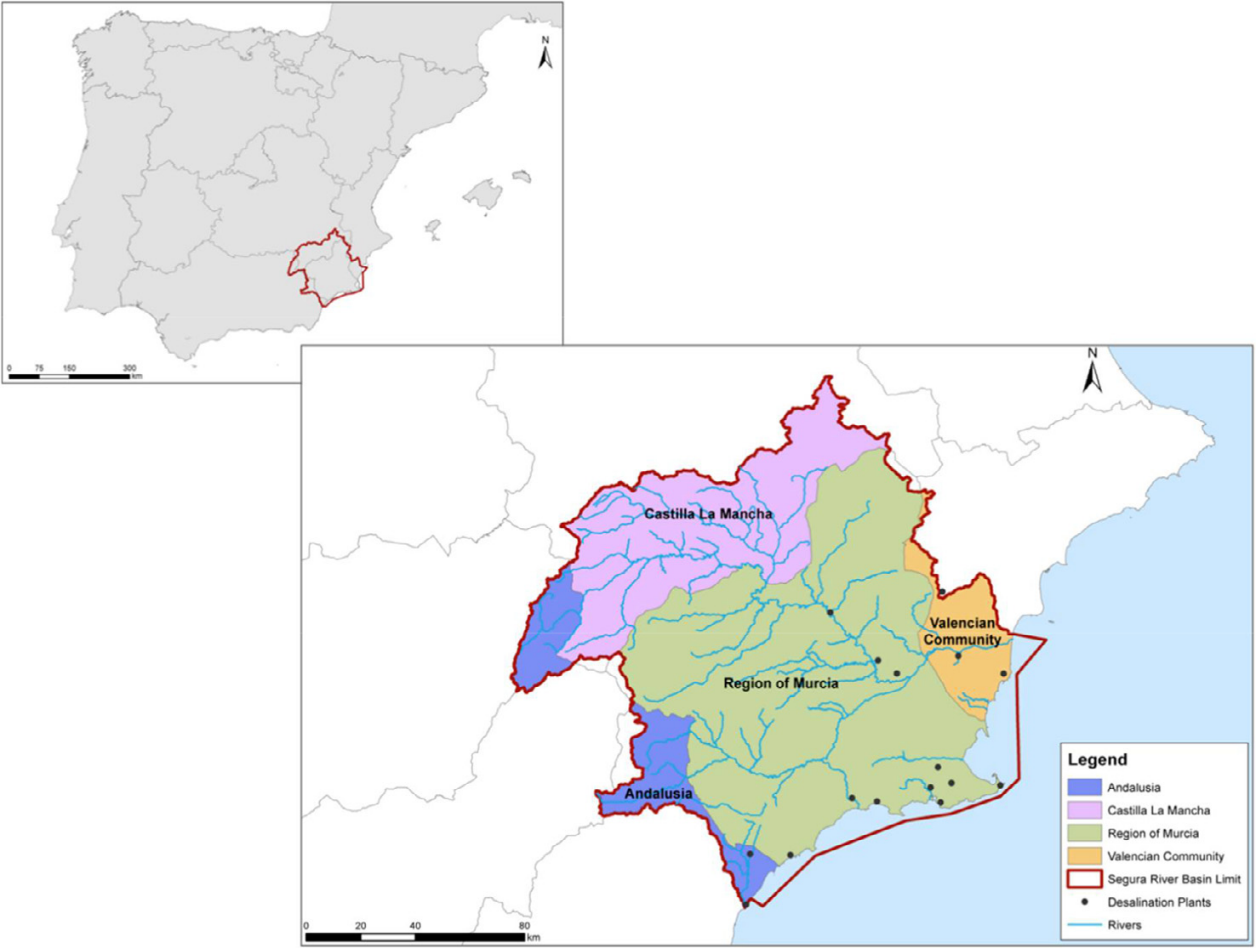
The watersheed of Segura: boundaries of the case study and desalination plants.

The data consists on the geographic information, distance and payload distance analysis and the environmental indicators (eleven ILCD midpoint categories and their Service Life Ratio) (available at Mendeley repossitory http://dx.doi.org/10.17632/z6db5w8d6k.1) concerning the recycling strategies analysed in [1] for the full-scale implementation of a recycling plant at the Segura´s watershed (Spain). Fig. 1 illustrates the boundaries of the case study and the desalination plants incuded int the studied region.

Table 1 includes the summary of the desalination facilities in the case study region obtained from AEDYR database. The main features included were the desalination capacity and the type of water treated (SW: sea water; BW: brackish water). This information allowed the estimation of the potential amount of EoL-RO generated by type en each facility.

**Table 1:** Inventory of desalination plants, capacity and type of water treated (SW: sea water; BW: brackish water).Source AEDYR database.

Location	Region	Facility name	Capacity (m^3^/day)	Water treated
Torrevieja	Valencia	Desaladora de Torrevieja	240,000	SW
Aguilas	Murcia	Águilas (Acuamed)	210,000	SW
Valdelentisco	Murcia	Valdelentisco	140,000	SW
Escombreras	Murcia	Escombreras	68,000	SW
Cartagena	Murcia	Canal de Cartagena	65,000	SW
Cartagena	Murcia	Canal de Cartagena Extension	65,000	SW
Cartagena	Murcia	Canal de Cartagena. San Pedro del Pinatar. I	65,000	SW
Cartagena	Murcia	Canal de Cartagena. San Pedro del Pinatar. II	65,000	SW
Aguilas	Murcia	Aguilas	20,800	SW
Aguilas	Murcia	Aguilas	16,000	SW
Mazarron	Murcia	CR Mazarron	13,500	BW
Almería Pulpi	Andalucía	Almería Pulpi	10,500	SW
Jacarilla	Valencia	Jacarilla - Alicante	8,750	BW
Cartagena	Murcia	Ampliación Refineria Repsol	8,400	SW
Cabo de Palos	Murcia	Arco Sur	7,000	BW
Almería Pulpi	Andalucía	Almería Pulpi	6,000	SW
Murcia	Murcia	IDAM LA MARINA	5,000	SW
Murcia	Murcia	Copisa El Pozo	4,000	BW
Cartagena	Murcia	Cartagena	3,600	SW
Murcia	Murcia	Murcia	2,800	BW
Cartagena	Murcia	Cartagena	2,700	BW
Cartagena	Murcia	Cartagena	2,700	BW
Murcia	Murcia	Agrícola Escucha	2,700	BW
Campo Cartagena	Murcia	Campo Cartagena	2,000	SW
Murcia	Murcia	Murcia (Finca Torremolino)	1,800	BW
Campo Cartagena	Murcia	Campo Cartagena	1,750	BW
Murcia	Murcia	Murcia (Agrohispaner)	1,175	BW
Abarán	Murcia	Abarán	1,080	BW
Campo Cartagena	Murcia	Campo Cartagena	1,000	SW
Villaricos	Andalucía	IDAM Covisa	1,000	BW
La Palma Mur	Murcia	La Palma Mur (Fejima)	600	BW
Murcia	Murcia	Murcia	600	BW
La Palma Mur	Murcia	Finca lo Triviño	500	SW
Campo Cartagena	Murcia	Campo Cartagena (Finca El Pasico)	330	BW
Hondón	Valencia	Hondón (Alicante)	250	SW
Molina de Segura	Murcia	Golosinas Fini	225	BW
Murcia	Murcia	Murcia	220	SW
Murcia	Murcia	Murcia Hero	190	BW
Murcia	Murcia	Murcia	100	BW

**Fig. 2:**
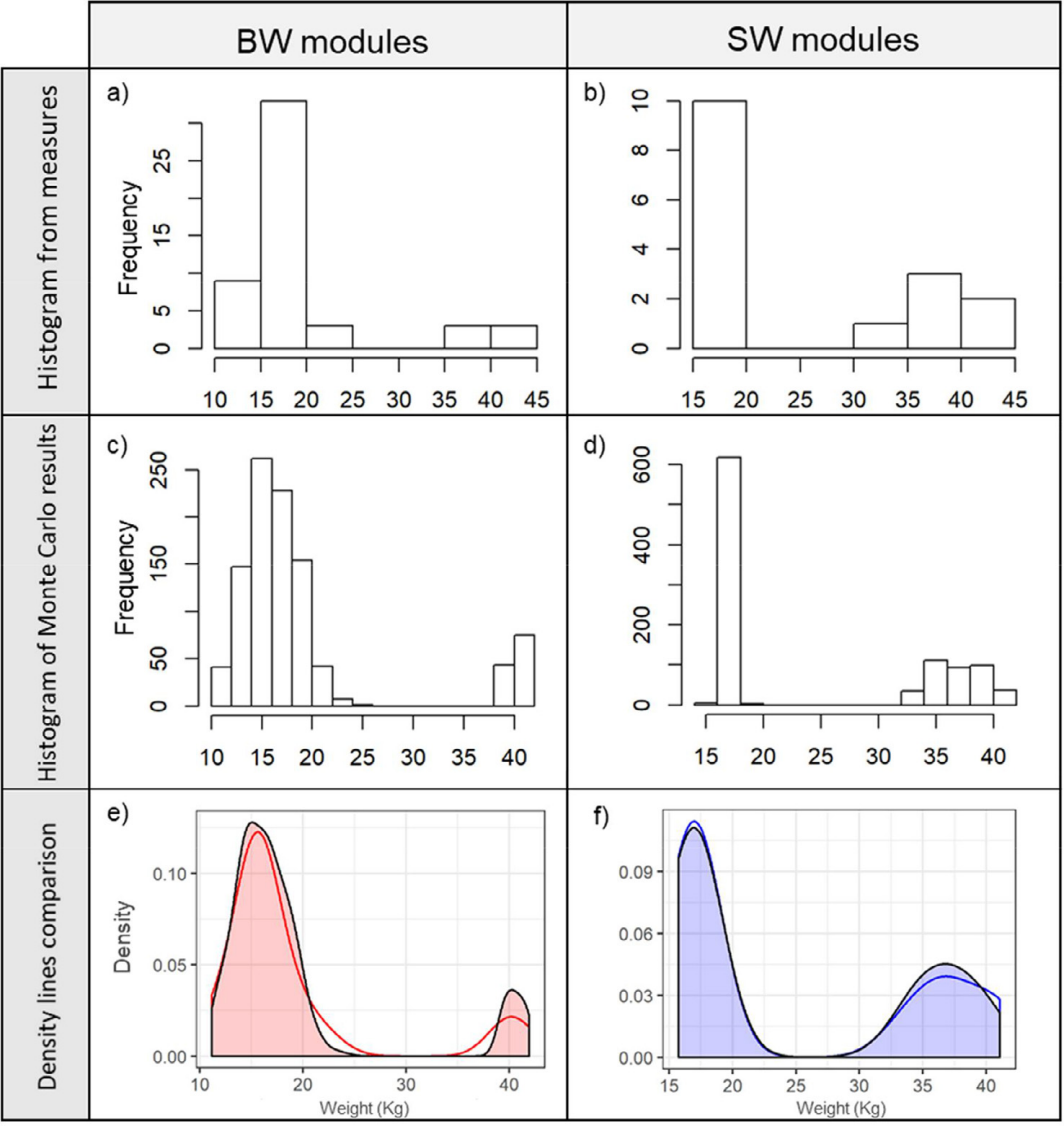
EoL-RO modules weight histograms for BW (a) and SW (b); Histogram of modelled weights by Monte Carlo results from 1,000 runs for BW (c) and SW (d); and comparison of density curves between the modelled and the real measures for BW (e) and SW (f). The adjust values (in e and f) are different to correct the different number of individuals.

The weights of the modules generated by the waste was extrapoted from the real measurement of 67 modules of different plants and the target water of the design (WD; if they were design to treat seawater (SW) or brackish water (BW)). Raw data can be found in the mendeley repository (DOI: 10.17632/z6db5w8d6k.1) in the file Modules weight.xlsx. Table 2 describes the fitted distributions from original measures of weights with the methodology described in section 2. 1. Fig 2 illustrates i) the results of the measurement (Fig 2a and Fig. 2b), ii) the Monte Carlo simulations projected by type (Fig 2c and Fig. 2d) in histrograms generated with R v3.4, and iii) the density functions of both generaterd with *ggplot 2 package* of the same software.

**Table 2:** Summary of modules weight and distribution parameters by water target of design (WD) (SW: sea water; BW: brackish water).

WD	Total Number(n)	Modules >=25 kg(n)	Modules <25 kg(%)	Modules >=25 kg(%)	Distribution < 25 kg	Distribution >= 25 kg
BW	51	6	88.2	11.8	Normal (15.95, 2.515)	Uniform (39–42)
SW	16	6	62.5	37.5	Normal (16.95, 0.37)	Uniform (33.4–41)

**Fig. 3:**
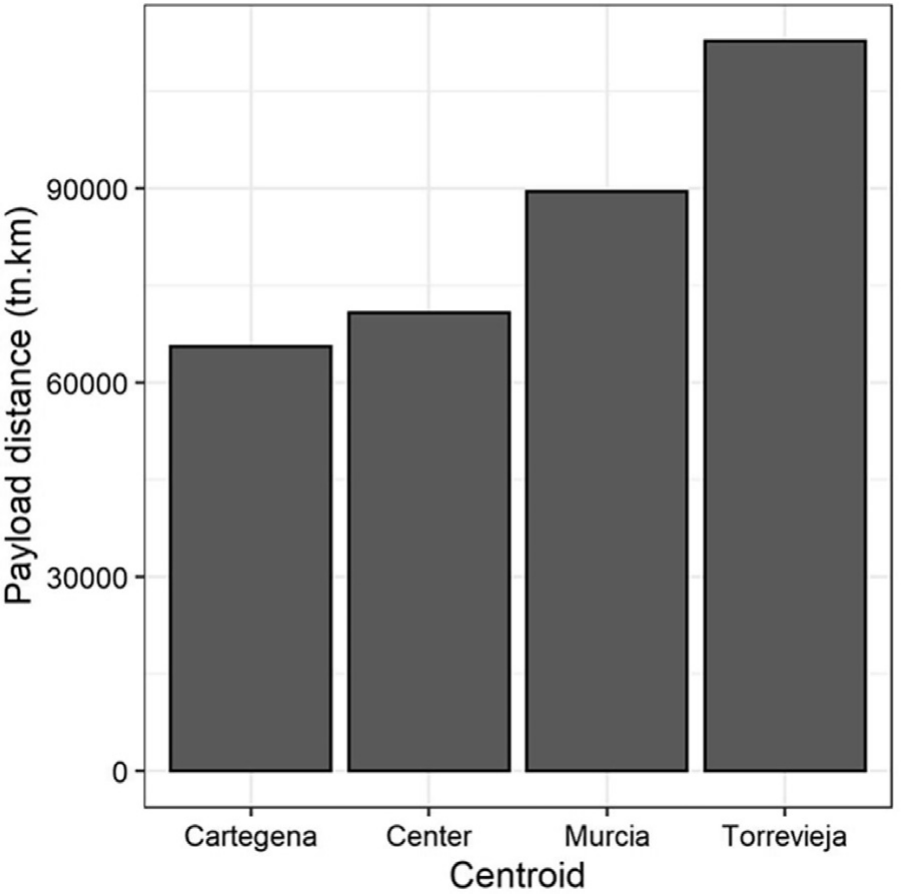
Total payload distance per centroid. Source data at repository.

The results for the four locations analysed are in the file RL_dib.csv at Mendeley dataset. It includes the resuls of the payload distance of each centroid with the expected waste stock estimated by the desalination capacity. The overall results are illustrated in the Fig 3. On the other side, The file Distances_dib.csv of the repository contents the logistic analysis results of the distribution of commercial and recycled modules. It includes the distances and payload distances by transport medium and the geographical information of locations.

Finally, the Life Cyce Impact Assessment and Service Life Ratio (SLR) results are at the respository in the file MC_prm_dib.csv. The Monte Carlo raw results and the summary in sum_prm_dbi.csv. Those results are aggregated around the functional unit of one recycled module at the secondary user location. In addition, the results were estimated with a secondary functional unit of the management of all the modules of Segura´s Watersheed. The files MC_pw_dib.csv and sum_pw_dbi.csv of the repository include the Monte Carlo results and the summary, respectively. In both files the results include the analysis of different strategies called Com and BW that differ on what type of modules are recycled. Com represents the management of all the modules (SW and BW) and BW just the recycling of BW modules.

## 2 Experimental Design, Materials and Methods

### 2.1 Reverse logistics analyses and plant location

The recycling plant location was defined through the criteria of the minimum payload distance with the following methodology. A first assessment was performed among the centroids of four suitable areas. To estimate the payload distances the desalination plants and their capacity of the area were identified (Table 1) to estimate the EoL-RO stock according to [2]. The distances were estimated by the shortest route with Google Earth roads in QGIS v3.8. Secondly, the modelling of EoL-RO module weights was performed with the experience of previous experimentations within Life-TRANSFOMEM project (http://www.life-transfomem.eu/). Fitdistrplus R package was used for fitting the weight distribution. The results were showed in Table 2. The centroid of Cartagena was chosen as the best option due to the lower payload distance (Fig 2) for further steps.

### 2.2 Comparison of distribution impact

For the comparison of the distribution impacts between recycled and new produced membranes three regions were defined related to the recycling plant location: regional, Iberian and European. Within these regions, 1,000 points per region were randomly obtained from ArcGIS v14. The selection of different functions available in different softwares was mainly defined by easiness and the software availability. In this case, it was a punctual use of a tool of ArcGIS v14 that was considered more practical. The comparison with the commercial distribution schemes was performed with two facilities in Germany and America. Euclidean distances were estimated and corrected by detour factor: 1.25 for road transport and 1.5 for shipping according to [3]. In the case of the transport from the American Facility, the closest docks were selected by end-user point (Table 3).

**Table 3:** Principal docks of Europe and their geographical coordinates.

Name	X	Y
Valencia Port	-0.325009508	39.444274513
Algeciras Port	-5.439506287	36.124952104
Barcelona Port	2.162315633	41.350440512
Sines Port	-8.845309387	37.937172398
Rotterdam Port	4.144285591	51.944733094
Antwerpen Port	4.407435865	51.240416294
Hamburg Port	9.966844272	53.506284553
Bremenhaven Port	8.546270031	53.576052673
Piraeus Port	23.591384182	37.957916864
Gioia Tauro	15.907300261	38.454453679
Ambarli Port	28.680360321	40.969163461
Le Havre Port	0.148985811	49.472485208
Genoa Port	8.880227186	44.409939478
La Spezia Port	9.844891589	44.109712215
Mersin Port	34.646849531	36.804510867
Gdansk Port	18.708933352	54.385977389
Marseille Port	5.337508581	43.349267857

### 2.3 Goal and Scope

The goal of the LCA was the assessment of the recycling implementation. System boundaries and scope were defined in [1]. The data was aggregated around the functional unit of the one EoL-RO module recycled. This funcitional unit was chosen to increase the comparability with other previous studies focus on the alternative end-of life options and recycling processes. Nonetheless, a secondary functional unit was also used for the Life Cycle Impact assessment: the recycling of all the EoL-RO modules of the Segura´s watershed generated in one year. This secondary functional unit evaluates the overall impact of the strategy. It allows the quantification of the impact of the strategy and the recycling in a macro scale allowing the comparison with other recycling activities or potential policies.

### 2.4 Life cycle impact assessment and service life ratio

The Life Cycle Impact Assessment was performed with OpenLCA v1.10 and R v3.4. The impact method ILCD-midpoint v.1.05 (OpenLCA/NEXUS) was used. Midpoint categories were used to evaluate the direct effect to the environment of the alternatives (Table 4). In particular ILCD-midpoint categories provide a wide vision of the main environmental concerns with high degree of reliability. Also, the service Life Ratio was estimated following [4].

**Table 4:** The ILCD-Midpoint v1.0.5 method categories and abbreviations.

Abbr.	Characterisation methods	Reference unit
A	Acidification	mol H+ eq.
GWP	Climate change (100 years)	kg CO_2_ eq.
FE	Freshwater eutrophication	kg P eq.
ME	Marine eutrophication	kg N eq.
HT, c	Human toxicity, cancer effects	CTUh
HT, nc	Human toxicity, non-cancer effects	CTUh
IR-e	Ionising radiation-ecosystems	CTUe
IR-hh	Ionising radiation-human health	kg U_235_ eq.
TE	Terrestrial ecotoxicity	mol N eq.
ET, f	Freshwater ecotoxicity	CTUe
OD	Ozone depletion	kg CFC-11 eq.
PCOF	Photochemical ozone formation, human health	kg NMVOC eq.
PM	Particulate matter/Respiratory inorganics	kg PM _2.5_ eq.
LU	Land use	kg C deficit
RD, f + m	Resource depletion, mineral, fossils and renewables	kg Sb eq.
RD, w	Resource depletion water	m^3^ water eq.

## Ethics Statement

 

## Declaration of Competing Interest

Eloy García-Calvo, Raquel García-Pacheco and Junkal Landaburu-Aguirre, co-authors, are also inventors of the Spanish Patent PCT/EP2016/30931 (08 July 2016): Transformation of spiral wound polyamide membranes after its industrial lifespan.

The authors declare that they have no known competing financial interests or other personal relationships which have, or could be perceived to have, influenced the work reported in this article.
